# Mechanisms underpinning effective peer support: a qualitative analysis of interactions between expert peers and patients newly-diagnosed with bipolar disorder

**DOI:** 10.1186/1471-244X-12-196

**Published:** 2012-11-09

**Authors:** Judith G Proudfoot, Amisha Jayawant, Alexis E Whitton, Gordon Parker, Vijaya Manicavasagar, Meg Smith, Jennifer Nicholas

**Affiliations:** 1 Black Dog Institute and School of Psychiatry, University of New South Wales, Hospital Road, Randwick, NSW, Australia; 2 School of Social Sciences, University of Western Sydney, Penrith South, NSW, Australia

**Keywords:** Bipolar disorder, Peer support, Experiential knowledge, Social support, Social comparison, Helper therapy

## Abstract

**Background:**

The increasing burden on mental health services has led to the growing use of peer support in psychological interventions. Four theoretical mechanisms have been proposed to underpin effective peer support: advice grounded in experiential knowledge, social support, social comparison and the helper therapy principle. However, there has been a lack of studies examining whether these mechanisms are also evident in clinical populations in which interpersonal dysfunction is common, such as bipolar disorder.

**Method:**

This qualitative study, conducted alongside a randomized controlled trial, examined whether the four mechanisms proposed to underpin effective peer support were expressed in the email exchange between 44 individuals newly-diagnosed with bipolar disorder and their Informed Supporters (n = 4), over the course of a supported online psychoeducation program for bipolar disorder. A total of 104 text segments were extracted and coded. The data were complemented by face-to-face interviews with three of the four Informed Supporters who participated in the study.

**Results:**

Qualitative analyses of the email interchange and interview transcripts revealed rich examples of all four mechanisms. The data illustrated how the involvement of Informed Supporters resulted in numerous benefits for the newly-diagnosed individuals, including the provision of practical strategies for illness management as well as emotional support throughout the intervention. The Informed Supporters encouraged the development of positive relationships with mental health services, and acted as role models for treatment adherence. The Informed Supporters themselves reported gaining a number of benefits from helping, including a greater sense of connectedness with the mental health system, as well as a broader knowledge of illness management strategies.

**Conclusions:**

Examples of the mechanisms underpinning effective peer support were found in the sample of emails from individuals with newly-diagnosed bipolar disorder and their Informed Supporters. Experiential knowledge, social support, social comparison and helper therapy were apparent, even within a clinical population for whom relationship difficulties are common. Trial registration number ACTRN12608000411347.

## Background

Management of chronic mental illness places a substantial strain on mental health resources, with population-based studies indicating that less than one-third of mental health needs are met [1]. In light of this, there has been an increasing interest in the use of peer-delivered services, the most well-known of which is the peer support group. Peer support produces a number of positive effects (for a review see [2]). Attendance at support groups is associated with improvements in symptoms, increases in participants’ social networks, better quality of life, reduced hospitalisations, improved coping, greater acceptance of illness, improved medication adherence, lower levels of worry, greater satisfaction with one’s own health and improved daily functioning [2]. Peers can also be involved as guides to patients receiving individual psychotherapeutic interventions. In contrast to the type of peer-to-peer support available through groups, support offered in individual interventions is typically provided by Informed Supporters - individuals who share a similar condition with patients, are able to successfully manage their illness and have been trained by a mental health professional to provide support [3].

### Mechanisms underpinning the benefits of peer support

The use of Informed Supporters in individual interventions has not been well studied, though there are several theoretical reasons why the involvement of expert peers in this context may be beneficial. Firstly, Informed Supporters are able to provide advice that is grounded in experiential knowledge, which is typically more practical in nature than advice based solely on clinical knowledge [4,5]. Reflecting their first-hand experience, Informed Supporters may be in a better position than clinicians to identify and address psychosocial issues, share their experience and convey information more clearly using “patient talk” - information conveyed using lay terms, actual feelings and personal experiences, rather than medical terminology [6]. Thus, Informed Supporters may be able to provide assistance in the form of easily understandable, practical strategies, and so help newly-diagnosed patients navigate the lifestyle changes required to effectively manage their illness.

Patients may also view peers as valuable in helping to address their psychosocial problems. In a study of telephone-based peer support for individuals with cancer, peers were consulted more about psychosocial and day-to-day issues (such as the impact of the illness on family and friends) than about issues relating to the medical treatment [7]. It has been suggested that patients may view peers as having a specific kind of knowledge, grounded in experience, that makes them especially adept at addressing psychosocial issues [7].

A second mechanism underpinning the benefits of peer support is social support. Social support has been defined as information leading a person to believe that he or she is cared for, loved, esteemed and valued, and that he or she belongs to a network of communication and mutual obligation [8]. The benefits of social support operate via two mechanisms: buffering against the adverse effects of stress, and promoting positive health behaviours (for a review see [9]). In the face of a stressor, social support from others may provide practical solutions that directly address or remove the source of the stress, or they may be able to provide emotional relief from the stress through empathic concern and distraction.

Receiving social support is associated with a number of positive health outcomes (for reviews see [10,11]). These include facilitation of adjustment to chronic physical and mental health conditions [5,12,13], reduced psychological distress [14,15] and greater perceived [16] as well as actual physical and mental health [9,17].

Social comparison theory proposes a third mechanism by which Informed Supporters may benefit those they are helping. ‘Social comparison’ is defined as the process of relating information about one or more other people to the self [18]. Drawing similarities between the self and others is one way of using this information, and is a process by which an individual can form a sense of identity within a group of similar others. Using social information in this manner has been shown to be beneficial for individuals with schizophrenia, where group identification was found to exert a protective effect on self-stigma, while enhancing self-esteem and self-efficacy [19].

Making an ‘upward social comparison’ is another way of using such information, and chronically unwell individuals who have regular contact with expert peers may feel an increased sense of hope and motivation as a result of comparison with a similar other who is effectively managing their illness. Similarly, given that Informed Supporters have been recipients of mental health services, they also serve as good role models for newly-diagnosed patients, many of whom may be encountering mental health services for the first time. Studies have shown that the strategies used by individuals who effectively manage their illness are similar to the strategies taught in most psychotherapy interventions [20]. Therefore, having contact with an Informed Supporter allows a patient the unique opportunity of observing someone successfully managing their illness through the use of strategies that are also taught by clinicians. Observing Informed Supporters using such strategies may result in positive behavioural change on the part of the supported patient, may subsequently increase their engagement with treatment, and help facilitate positive relationships with mental health service providers [21-23].

Lastly, the Informed Supporters themselves may also gain a number of benefits from helping others manage their illness, a phenomenon known as the helper therapy principle [24]. According to Skovholt [24], the benefits typically derived from helping others include an enhanced sense of interpersonal competence from impacting on another’s life, the ability to receive personalised feedback from working with others and an improved sense of self and personal value that results from the social approval received by those helped.

A number of studies provide support for the notion of helper therapy. A survey of 3617 elderly people revealed that helping others was associated with a greater sense of personal control and lower levels of depression [25]. In a study of abused women, those who gave peer support viewed their ability to provide support as indicative of their own recovery [26]. Additionally, studies have found that peer supporters view their role as improving their communication skills and self-confidence, creating a greater appreciation for the emotional states of other people [27], enhancing their applied knowledge [28], perceptions of social support [29], perceived health, wellbeing [30] and quality of life [31]. Helping others also serves to increase the helper’s social role repertoire, a factor that has been shown to have positive effects on health [32]. Some researchers have even suggested that the beneficial effects of helping others may be substantial enough to consider helping as possible adjunct to treatment for a disease [33].

### Peer support in the treatment of bipolar disorder

Bipolar disorder is a severe psychiatric illness that is characterised by fluctuating periods of depression and hypomania/mania. Pharmacotherapy is the principal treatment for the disorder, however, poor compliance with medication means that relapse rates are high, with major relapses typically occurring every 17-30 months [34]. Medication compliance is often enhanced when psychoeducation is provided alongside pharmacotherapy [35], with online psychoeducation programs becoming increasingly popular. Such interventions are typically offered as stand-alone programs or with support from mental health professionals, with evidence indicating superior outcomes, greater compliance and decreased rates of attrition in supported, compared to unsupported programs [36-38].

However, despite the benefits of peer support observed in other chronic conditions, there are reasons why peer support may be less effective in individuals with bipolar disorder. First, bipolar disorder is often associated with marked difficulty in establishing and maintaining relationships [39-41]. Individuals with bipolar disorder have poorer marital outcomes [42,43], fewer social interactions [44], poorer quality friendships [45] and heightened conflict with peers, family members and work colleagues [46], relative to healthy controls. Interpersonal dysfunction is even evident in the early stages of the disorder, where affective dysregulation characterised by frustration and anger has been documented in interpersonal situations in adolescants with bipolar disorder [47]. Such interpersonal difficulties associated with bipolar disorder may potentially limit the benefits typically gained in supported interventions, as peer support requires the formation and maintenance of a relationship between the Informed Supporters and those they support.

Second, providing peer support can place a high burden of work and responsibility on supporters, it can expose supporters to emotionally-charged and often blurred relationships with those they are supporting, and it can be inconvenient when paired with the burden of managing their own illness [48]. Such factors may be especially problematic for individuals with bipolar disorder, as exacerbation of mood episodes have been closely linked with life stress, and particularly the stress associated with emotionally-charged interpersonal relationships (for a review see [49]).

Last, social support, one of the mechanisms underpinning effective peer support, has been shown to have less impact on resilience to life stress in bipolar disorder than in other populations. For example, in a study examining the effects of social support on symptom frequency and duration, social support was found to reduce episode duration and also to lower vulnerability to depression, however, it did not buffer against the effects of life stress [50]. This is in contrast to the positive effects of social support that have been observed for other chronic conditions, such as unipolar depression [12] and schizophrenia [19]. The authors suggest that individuals with bipolar disorder may require more intensive social support, given that the bidirectional relationship between life stressors and bipolar symptomatology may exacerbate and perpetuate stress [40].

The aim of the current study was to examine, using a qualitative approach, whether the mechanisms underlying beneficial peer support emerged in the interactions between patients with newly-diagnosed bipolar disorder and their Informed Supporter. To our knowledge, this is the first study to examine whether these four mechanisms (namely, experiential knowledge, social support, social comparison and helper therapy) were present in a sample of individuals with bipolar disorder. The research is therefore important in determining whether peer support may be a suitable addition to psychological interventions in this population.

## Method

### Design

This qualitative study was an adjunct to a randomized controlled trial (RCT) that evaluated the effectiveness of a web-based psychoeducation program delivered with and without online peer support to individuals recently diagnosed with bipolar disorder [3,51,52]. Results revealed greater treatment adherence in the supported group compared to the unsupported groups, although improvements in clinical symptoms were no greater in the supported group, in contrast to studies of supported online interventions for high prevalence conditions [53-55].

### Participants

Participants who had been diagnosed with bipolar disorder in the previous 12 months were recruited to the RCT through the Black Dog Institute clinic and website, via media advertisements and flyers distributed to general practitioners and psychiatrists, as well as through information that was distributed to community mental health organisations across Australia. Inclusion criteria were aged 18 years or above; had received a diagnosis of bipolar disorder by a GP or psychiatrist within the last 12 months; were receiving on-going management for their bipolar disorder by a health professional; had access to the internet, email and a printer; had adequate levels of computer literacy; were able to read and write English; resided in Australia; had received a score of 22 or more on the Mood Swings Questionnaire [56] to confirm diagnosis. An additional inclusion criterion for the qualitative study was that they had received email support from an Informed Supporter within the RCT. The Informed Supporters were recruited through the Black Dog Institute, a clinical and research institute specialising in mood disorders. Inclusion criteria were that they had been diagnosed with bipolar disorder by a psychiatrist and that they had been assessed by their treating psychiatrist as having effectively managed their condition for at least two years prior to the study. All participants provided informed written consent to participate in the study.

### Intervention

Participants in the RCT were allocated to one of three conditions: (1) Online Bipolar Education Program; (2) Online program plus email support from an Informed Supporter; and (3) a control condition consisting of weekly emails with links to simple information about bipolar disorder [51].

Participants in the two intervention conditions received one online module per week over eight weeks. Those with email support also received 1-2 emails per week from an Informed Supporter. The Informed Supporters were paid for their time in the study (including email support, training and supervision). Their support was entirely via email and was designed to answer questions from participants, as well as to provide participants with examples of how to apply the strategies covered in the online modules to their everyday lives. There was no limit on the number of emails sent by participants, but emails to a participant from an Informed Supporter were restricted to two 300-word emails per week.

Questions of a clinical nature were referred to the Black Dog Institute clinicians for response. All emails sent by Informed Supporters to participants were copied to the research team for checking and quality assurance, while emails from RCT participants were monitored for ‘red alerts’ (severely low or high mood or any adverse events). In the event of a red alert, an email was sent to advise the participant to contact their health practitioner immediately. The Informed Supporters were trained for the role by the first and fifth authors, and they attended monthly supervision sessions to discuss issues and concerns raised by participants, and to monitor their adherence to the research protocol.

### Procedure

To explore whether the proposed mechanisms of peer support emerged in the email interchange between the RCT participants and their respective Informed Supporters, a selection of the emails was collated and qualitatively analysed. In addition, the Informed Supporters were invited by phone to take part in face-to-face interviews carried out by the second author, a female graduate medical student who was trained in qualitative interview techniques and supervised by the first author. Participants had no relationship with the interviewer prior to the commencement of the study, though they were told that the interviewer was a medical intern and was interested in their experience of being an Informed Supporter. A semi-structured interview schedule was developed, with 15 questions covering three major areas: general questions regarding the Informed Supporter role (e.g., “What motivated you to become and Informed Supporter?), specific questions regarding the Informed Supporter role (e.g., “What impact do you think an Informed Supporter has on participants?”), and questions about the impact of the Informed Supporter role on their own bipolar disorder (e.g., “Has being an Informed Supporter impacted in any way on your own condition?”). The interviews were conducted at the Black Dog Institute in a one-on-one manner over a single, one-hour session, and were recorded using a Dictaphone with informed written consent. After the interviews, the transcripts were sent to the Informed Supporters to be validated or edited as they felt appropriate. The analysis of the email transcripts was carried out retrospectively, after the completion of the RCT, and therefore it was not possible to get feedback on the qualitative study from participants. However, all participants were offered individualised feedback on the questionnaires they completed for the RCT.

All components of this study were performed in accordance with the Declaration of Helsinki and ethics approval was obtained from the Human Research Ethics Committee of the University of New South Wales.

### Analysis

**Figure 1:**
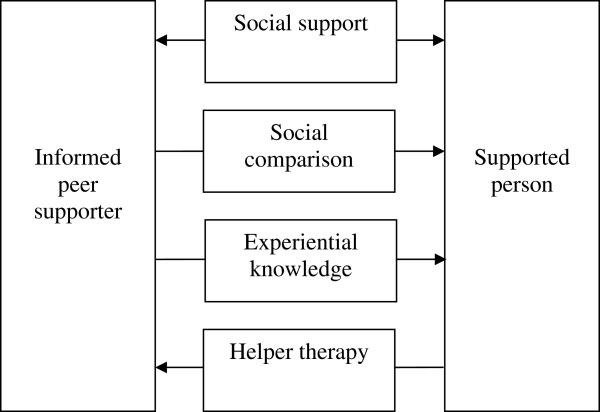
Directionality of the mechanisms underpinning effective peer support.

Participants’ emails and the Informed Supporter interview transcripts were analysed and coded thematically using a directed content analysis approach. In this framework, existing theory or research provides a method of structuring the analysis process, whereby researchers use key concepts or variables established in past research to code text responses. This was deemed to be the most appropriate form of qualitative analysis for the present study, as it allows the researcher to map existing theoretical principles onto the beliefs, values and attitudes of participants [57]. Text from the interview transcripts and email interchange was initially coded for the four theoretical mechanisms proposed to underpin the benefits of peer support. These were: advice grounded in experiential knowledge, social support, social comparison, and helper therapy (shown in Figure 1). Instances where benefits of experiential knowledge were demonstrated were defined as: participants referring to the value of their Informed Supporter sharing their past experiences; participants enquiring about the subjective and psychosocial aspects of dealing with the disorder and what it was like to live with it; instances where Informed Supporters expressed feeling that their own experience had given them a unique ability to assist the participants; instances where the Informed Supporters commented that their past experience had helped them to relate to and understand the experiences of those they were supporting. Instances where the benefits of social support were demonstrated were defined as: when appreciation of the social connectedness offered by the peer support relationship was expressed; emotional support was offered or received (i.e. reassurance, normalisation, decreasing feelings of isolation); instrumental support was offered or received (i.e. assistance with problem-solving). Instances where social comparison was demonstrated were defined as: where the resilience and success of the Informed Supporters managing their illness was referred to as motivating or inspiring the participants; when the Informed Supporters were promoting positive behaviours or attitudes in the participants by modelling resilience, active illness management strategies and good relationships with mental health service providers. Finally, evidence of the benefits of helping (i.e. the helper therapy phenomenon) was defined as: times when the Informed Supporters reported positive outcomes as a direct result of helping their RCT participants (e.g. through a sense of achievement, through their enhanced understanding of illness management strategies).

Consistent with standard qualitative methods, we sampled to saturation from the emails of the 134 participants who received the online psychoeducation program with adjunctive peer support within the RCT, and from the interviews with the Informed Supporters. A total of 104 segments of text were extracted from emails between 44 participants and 4 Informed Supporters, as well as from interview transcripts from 3 of the Informed Supporters. The text segments were coded by the first and third authors who were blind to each others’ coding. A specialist software package was not used for the collating and coding. An initial inter-rater reliability of 0.9 was achieved, with an agreement on 97/104 segments of text. Discrepancies over the other seven segments of texts were then resolved through discussion between the raters. Rich textual examples were extracted to highlight the way in which each of the mechanisms manifested in the current sample. The study adhered to the consolidated criteria for reporting qualitative research (COREQ) checklist [58].

## Results

Within the RCT, the number of emails sent by participants in the group receiving the online psychoeducation program with adjunctive peer support (n = 134, 73.9% female) ranged from 0 to 17, with 12.7% (n = 17) having sent no emails, 15.7% (n = 21) having sent only one email, and 71.6% (n = 96) having sent two or more emails. In this group, the average number of emails sent was 4.3 (SD = 3.8). Within the qualitative study, the number of emails sent by the sample of 44 participants (75% female) ranged from 1 to 15, with 4.6% (n = 2) sending only one email and 95.5% (n = 42) sending two or more emails. The average number of emails sent was 6.5 (SD = 3.7).

Of the four Informed Supporters who were involved in the study, three took part in face-to-face interviews: two males (aged 54 and 59 years) and one female (aged 28 years). The fourth Informed Supporter was not available.

Examples of all four peer support mechanisms were found in the qualitative data. Of the 104 text examples coded, 48.1% (n = 50) were categorised as advice grounded in experiential knowledge, 30.8% (n = 32) as social support, 6.7% (n = 7) as social comparison and 14.4% (n = 15) as helper therapy. The following are examples from the email interchange and interview transcripts, which express in rich detail the way in which the four mechanisms operated in the current sample.

### Advice grounded in experiential knowledge

The email interchange revealed that many of the newly-diagnosed patients were interested in gaining a sense of what it was like to live with bipolar disorder.

*“I have many questions which have to do with the experience more than the clinical side of things.”* [Participant #10 (female, aged 40-49) reply to Informed Supporter #3 (female, aged 28)]

They expressed a need for practical strategies to help them cope with the symptoms and medication side effects, and the Informed Supporters were able to offer this assistance in a way that was both practical and empathic.

*“With regard to your sense of the drug haze, I know it well, for me the point was to find a combination of drugs that didn’t leave me feeling like a zombie. I know the dilemma well, needing to control the labile swings and yet not appreciating the fog it leaves you in for most of the day…try taking your medication when it least affects your activity.”* [Informed Supporter #2 (male, aged 59) reply to RCT participant #42 (female, aged 18-29)]

The Informed Supporters also reported that their own experiences gave them a unique perspective on bipolar disorder.

*“… otherwise it’s only explained by doctors and clinicians, and they have a very specific view of the whole thing, and therefore their approach to it is technically orchestrated, whereas we’re not. We’re coming from a point of view of sensation, of feeling. So for me, it’s all about feelings, it’s all about the things that we go through as bipolar people, rather than the clinical understanding of it.”* [Informed Supporter #2 (male, aged 59)]

Along with this, came the sense from Informed Supporters that they had an ability to communicate with the participants in a way that may differ from health professionals.

*“… if I wanted to give them practical advice on things that they could do, I could maybe present it in a way that someone else couldn’t. Because I remember how it felt. So I can present it in a way that they can understand, or it won’t be too scary for them….I can communicate with them, like maybe someone without the experience can’t.”* [Informed Supporter #3 (female, aged 28)]

### Social support

The benefits of social support were discussed in a number of email exchanges, with some participants saying to their Informed Supporter that they were their only form of support.

*“Thanks for the time and effort you are spending being a support person. I really appreciate it. It feels like the only link I have with any form of support.”* [Participant #272 (female, aged 40-49) reply to Informed Supporter #1 (male, aged 54)]

*“It was heartening when I did finally check my emails to see so many concerned ones when I've felt very alone.”* [Participant #602 (female, aged 30-39) reply to Informed Supporter #2 (male, aged 59)]

The benefits derived from social support frequently were attributed to a sense of normalization surrounding the illness.

*“It feels strange to read your accounts and think ‘Wow, I'm not the only one!’* [Participant #30 (female, aged 18-29) reply to Informed Supporter #2 (male, aged 59)]

*“I guess the big revelation for me has been: I'M NOT ALONE…otherpeople experience this too!”* [Participant #282 (male, aged 30-39) reply to Informed Supporter #2 (male, aged 59)]

Such feelings of being more socially connected were also reiterated by the Informed Supporters.

*“Oh yes, there is great solidarity. Enormous solidarity. They’re like me, you know?”* [Informed Supporter #1 (male, aged 54)]

The Informed Supporters reported feeling able to overcome participants’ sense of isolation by opening up a channel of communication and revealing a level of understanding garnered by their first-hand experience of the disorder. As expressed by one Informed Supporter:

*“I really got the sense that the participants really needed that contact…they really wanted someone there to talk to.”* [Informed Supporter #2 (male, aged 59)]

This theme was also reflected by some of the participants.

*“How wonderful it is to talk with someone else with whom I can share a journey and understand openly and honestly with, without getting too embarrassed.”* [Participant #252 (female, aged 40-49) reply to Informed Supporter #2 (male, aged 59)]

### Social comparison

Social comparison between the RCT participants and Informed Supporters was evident in a number of the email exchanges, and appeared to have the effect of promoting hope and motivation on the part of the participants.

*“It's great that the bipolar doesn't affect you too much these days. There is hope then, I'm glad.”* [Participant #259 (female, aged 40-49) reply to Informed Supporter #1 (male, aged 54)]

*“… since being diagnosed I have not yet meet anyone that I know shares this disorder. It is nice just to know that someone does and that someone is living a normal life despite this diagnosis.”* [Participant #267 (female, aged 18-29) reply to Informed Supporter #2 (male, aged 59)]

There were several instances where social comparison appeared to have positive effects on the participants in terms of promoting faith in the effectiveness of treatment strategies.

*“I'm looking forward to CBT so I could accurately identify triggers to my behavior, much like you are able.”* [Participant #559 (female, aged 18-29) reply to Informed Supporter #4 (female, aged 29)]

The Informed Supporters responded by reinforcing the importance of maintaining relationships with health professionals, and of adopting a proactive and collaborative approach to treatment.

*“I personally have had the same doctor for all this time, and we, note WE, have changed medication 4 times over that 14 year period. I hasten to add that I have never been better/weller/happier, so I really am advocating becoming knowledgeable as you can about treatment options, and vigorously and democratically pursuing those options.”* [Informed Supporter #1 (male, aged 54) reply to participant #102 (female, aged 40-49)]

This extended to promoting adherence to medication.

*“I too felt like a guinea pig, but eventually with the help of my psychiatrist we found the right combination, it took time believe me. Hang in there and truly believe that the people helping you do know what they are doing, they have seen it all before, despite the fact that you feel the way you do.”* [Informed Supporter #2 (male, aged 59) reply to participant #86 (female, aged 18-29)]

The Informed Supporters reinforced the importance and effectiveness of stay well plans in managing mood episodes.

*“On that point, the interesting thing you said was that you fear the return of bad times…I know the feeling very well, it is a constant concern at first…that is until you begin to notice the level of distress is much less if in fact you maintain your medication and keep to your stay well plan you have laid out for yourself. I agree, it is a worry to think that you are going to deal with it all again but believe me the episodes are far less stressful when there is a plan of action.”* [Informed Supporter #2 (male, aged 59) reply to participant #24 (female, aged 40-49)]

### Helper therapy

The Informed Supporters appeared to gain a number of benefits from their role. These manifested as a greater awareness of strategies they could use in the management of their own condition.

*“It was actually helping me as well, because I was always rethinking the topic at hand and how best to deal with a certain situation. I’m always honing my own skills because even though my bipolar is manageable, it still has to be managed.…the main thing that it’s done is really teach me how much it’s an action-based recovery. Without the action, there is no recovery.”* [Informed Supporter #3 (female, aged 28)]

*“The fact that I am involved in something that is a part of my own well-being and sense of being. So this Informed Supporter program helps me do that because it keeps me on track. It keeps me thinking about my own health and wellbeing. The searching for explanations for my own illness, for my own feelings, the search for rationale for being who I am has developed through the Informed Supporter program. I’ve come to understand bipolar disorder, I suppose…a lot quicker because of this program.”* [Informed Supporter #2 (male, aged 59)]

The Informed Supporters also reported that their role had given them a greater sense of competence, and connectedness to the mental health system.

*“I’m far more competent now than what I was initially…I think I’ve probably grown emotionally from this as well.”* [Informed Supporter #1 (male, aged 54)]

*“I’m not battling this by myself, there’s a whole bunch of other people involved in making this work so I’m part of the cog…a cog in the machine. So that motivates me, to play a positive role.”* [Informed Supporter #2 (male, aged 59)]

The reciprocal relationship between the RCT participants and the Informed Supporters also appeared to have benefits for the Informed Supporters in terms of devising coping strategies.

*“… at least you know what has to be done to counter the effects of the triggers that affect your function…Some of the actions you are aware that must be taken are spot on…Well done, in fact I am going to implement some of those things for myself, thank you…”.* [Informed Supporter #2 (male, aged 59)] reply to RCT participant #28 (female, aged 18-29)]

## Discussion

This study explored the subjective experiences of individuals recently diagnosed with bipolar disorder who received an online psychoeducation program with adjunctive support from informed peer supporters. The aim of our qualitative study was to explore whether any of the mechanisms underlying the benefits of peer support in the management of chronic illness (experiential knowledge, social comparison, social support, and the helper therapy principle), emerged in the interactions between the participants and their peer supporters. To our knowledge, this is the first investigation of these factors in relation to the treatment of bipolar disorder. Our study is particularly interesting in that individuals with bipolar disorder are known to have difficulty in establishing and maintaining interpersonal relationships – a key mechanism through which peer support operates.

Advice grounded in experiential knowledge is typically pragmatic in nature. The practical strategies provided by the Informed Supporters complemented the information provided by the participants’ health professionals in assisting them to implement the lifestyle changes needed to effectively manage their illness. In the current study, participants asked their Informed Supporter for information on how to deal with the practical aspects of managing the illness, such as the side-effects of medication, how to inform others of their diagnosis, and how to deal with the impact of their bipolar disorder on their work and relationships. Many also voiced a desire to understand what it was like to live with the disorder long-term. The Informed Supporters were very helpful in addressing these concerns. They reported that having experienced bipolar disorder themselves gave them the ability to communicate practical strategies in ways that were meaningful to the participants, and served to highlight the role of an Informed Supporter in providing support and experience from a psychosocial and non-clinical perspective.

Two other mechanisms underpinning effective peer support that were examined in the present study were the motivational effects of participants comparing themselves to their Informed Supporter, and the positive impact of receiving additional social support. In the email interchanges, there were numerous examples of the participants expressing frustration with the mental health system or with their primary health professional. Changes to medication appeared to be a key source of this frustration, and after multiple medication changes, participants reported diminishing confidence in their ability to get well. In these situations, the Informed Supporters provided both a social support and a social comparison function.

First, the Informed Supporters provided emotional support and normalized the participants’ frustration. Their own experiences gave them the advantage of being able to sympathetically understand the participants’ experiences and illness. This type of understanding is rare in interactions between health professionals and their clients. While health practitioners are adept at giving information, many overlook psychosocial issues and the impact of the illness on the person [6]. When offered by a peer, sympathetic understanding may serve to decrease feelings of isolation, as well as the social distance between the client and the service provider. Such factors may have been particularly important for the participants in the current study, as the sense of isolation and stigma is known to be especially strong in the period of time immediately after diagnosis [52].

Secondly, by demonstrating their own competence in the management of their illness, the Informed Supporters were able to provide participants with direct evidence of the benefits of persisting with treatment regimes, thereby motivating participants to persist with their own treatment. Similar effects to those observed in the current research have been found in a study of individuals with schizophrenia, who showed an increased trust in psychiatric treatment following participation in a peer-supported psychoeducation program [59]. This suggests that one of the key advantages of using peer supporters throughout individual interventions, and in particular treatment programmes for bipolar disorder, is that they help individuals maintain trust in mental health services at crucial times where unsupported individuals may begin to disengage.

A final mechanism by which peer support exerts its benefits is via the positive effects it has on those providing the support – the helper therapy principle [60]. Peers involved in providing mental health services experience a number of benefits from helping, including increased confidence in their own capabilities, enhanced sense of control over their own illness, and increased empowerment and hope [2]. Helping others with a similar condition has also been found to improve quality of life in peer supporters [61,62]. Qualitative data from the present study suggests that the Informed Supporters gained a number of benefits from helping the participants throughout the psychoeducation program, including a greater sense of competence, a greater awareness of the factors that influenced their own bipolar disorder, improved knowledge of the strategies used to manage the disorder, as well as a sense of belonging to a wider community who were actively attempting to improve existing treatments. These findings indicate that even with a clinical condition that is associated with interpersonal difficulties, benefits associated with experiential knowledge, social support, social comparison and helper therapy can be found.

### Limitations

There are some limitations that must be kept in mind when interpreting the current data. First, whilst the use of a purposive sample of recently-diagnosed individuals allowed peer support mechanisms pertinent to this population to be explored through naturally-occurring email conversations, the sample may also have been very computer-literate and all had access to the internet. However, it has also been argued that the increasing internet access and usage by the general population may decrease this limitation [63]. Secondly, the study protocol restricting Informed Supporters to two emails per week may have limited the exploration of issues and thus the richness of the data collected. Lastly, due to resource restrictions, interviews were only conducted with the Informed Supporters and not with the RCT participants. Thus information as to why some participants did not engage fully with their Informed Supporter is lacking. It is therefore possible that the issues noted as possible threats to the effectiveness of peer support in bipolar disorder may have contributed to the underuse of the peer support service in a proportion of patients. Given that effective peer support is reliant on the patient engaging with their peer, it is important that future studies of supported interventions include an examination of factors that may contribute to poor engagement with peer supporters.

## Conclusion

The present study is the first to investigate whether the mechanisms proposed to underpin effective peer support were apparent in the email exchanges between individuals newly-diagnosed with bipolar disorder and their Informed Supporter, over the course of an online psychoeducation intervention. Rich examples of experiential knowledge, social support, social comparison and helper therapy were found in the interchange, despite the reported interpersonal difficulties associated with bipolar disorder.

## Competing interests

The authors declare that they have no competing interests.

## Authors’ contributions

JP, GP, VM and MS designed the RCT and the qualitative study, and GP, VM and MS wrote and developed the Online Psychoeducation Program for Bipolar Disorder. Informed Supporters were trained and supervised by JP and VM. AJ performed the face-to-face interviews with the Informed Supporters. AW and JP performed the qualitative analysis on the email exchange and Informed Supporter transcripts. AW and AJ drafted and edited the final manuscript under the supervision of JP, and received clinical advice and assistance with proofreading from JN. All authors reviewed and approved the final manuscript.

## Pre-publication history

The pre-publication history for this paper can be accessed here:

http://www.biomedcentral.com/1471-244X/12/196/prepub
